# Pulmonary Fibrosis Beyond the Lung: Adipose Tissue as a Systemic Modifier of Fibrotic Remodeling

**DOI:** 10.3390/diagnostics16101493

**Published:** 2026-05-14

**Authors:** Panagiota Tsiri, Bruno Crestani, Arnaud Mailleux, Argyrios Tzouvelekis

**Affiliations:** 1 Department of Internal and Respiratory Medicine, Medical School, University of Patras, 26504 Patras, Greece; tsiripanayiota@gmail.com; 2 Centre de Recherche sur l’Inflammation, Université Paris Cité, Inserm, 75018 Paris, France; bruno.crestani@aphp.fr (B.C.); arnaud.mailleux@inserm.fr (A.M.); 3 Service de Pneumologie et Transplantation, Centre de Référence Constitutif des Maladies Pulmonaires Rares, Assistance Publique-Hôpitaux de Paris, Hôpital Bichat, 75018 Paris, France; 4 Department of Pulmonary, Critical Care and Sleep Medicine, Yale School of Medicine, New Haven, CT 06511, USA

**Keywords:** pulmonary fibrosis, fibrotic ILD, adipose tissue, adipokines, biomarkers, metabolic reprogramming

## Abstract

Pulmonary fibrosis is increasingly viewed as a lung-centered disease influenced by systemic metabolic and inflammatory context. Adipose tissue, through its endocrine, paracrine, and immunometabolic functions, may act as a systemic modifier rather than a primary initiator of fibrotic remodeling. Epidemiologic and imaging studies link visceral adiposity, body composition, and adipose-related mediators such as leptin and IL-6 with subclinical interstitial abnormalities and outcomes in fibrotic ILD, while genetic and clinical data support a context-dependent protective role for adiponectin signaling. Mechanistic evidence suggests that adipose dysfunction may influence fibrotic remodeling through adipokine imbalance, inflammasome activation, perivascular inflammation, profibrotic macrophage polarization, epithelial maladaptation, cellular senescence, and fibroblast metabolic reprogramming. These pathways may converge to sustain myofibroblast activation and extracellular matrix deposition. Clinically, adipose dysfunction overlaps with common ILD comorbidities and may inform metabolic phenotyping, biomarker development, and patient stratification. Therapeutic implications remain investigational, with the strongest rationale supporting metabolic reprogramming strategies and biomarker-enriched studies rather than routine adipose-targeted therapy. Overall, the adipose–lung axis provides a framework for integrating systemic metabolic biology into the pathogenesis and clinical management of fibrotic ILD.

## 1. Introduction

Pulmonary fibrosis (PF) encompasses a heterogeneous group of interstitial lung disorders characterized by aberrant tissue repair, persistent fibroblast activation, and progressive extracellular matrix deposition, ultimately leading to irreversible architectural distortion, respiratory failure, and premature death [1]. Idiopathic pulmonary fibrosis (IPF), the most frequent and progressive fibrotic ILD, is associated with poor prognosis and a median survival of approximately 3–5 years after diagnosis [2]. Although antifibrotic therapies such as pirfenidone and nintedanib slow disease progression, and newer agents such as nerandomilast are expanding the therapeutic landscape, these treatments do not reverse established fibrosis [3,4,5].

Traditionally, fibrotic ILDs have been conceptualized as disorders driven primarily by repetitive epithelial injury, aberrant epithelial repair, and sustained fibroblast activation at the center of pathogenesis. However, increasing evidence suggests that pulmonary fibrosis is not solely a lung-restricted process but is influenced by systemic factors that modulate inflammatory tone, immune responses, and cellular metabolism. Among these, adipose tissue has emerged as a particularly relevant endocrine and immunometabolic organ capable of shaping fibrotic susceptibility across organ systems [6,7].

Adipose tissue is no longer viewed as a passive energy reservoir but as an active source of adipokines, cytokines, lipid mediators, and other bioactive signals that regulate metabolism, immune homeostasis, and tissue repair. In obesity and visceral adiposity, adipose tissue becomes dysfunctional, characterized by adipocyte hypertrophy, hypoxia, immune cell infiltration, and a shift toward a chronic pro-inflammatory secretory phenotype [8,9]. This dysfunctional state may alter systemic homeostasis in ways that favor maladaptive repair and persistent stromal activation.

Importantly, adipose dysfunction has already been implicated in fibrotic remodeling beyond the lung. In the liver, visceral adiposity and insulin resistance are central drivers of steatotic liver disease and progressive hepatic fibrosis. In the cardiovascular system, epicardial and perivascular adipose depots have been linked to myocardial fibrosis, vascular stiffening, and adverse remodeling. In diabetes and chronic kidney disease, adipose-associated metabolic inflammation contributes to renal fibrogenesis and end-organ injury [10]. Collectively, these observations support a broader paradigm in which dysfunctional adipose tissue acts as a systemic profibrotic modifier rather than an organ-restricted phenomenon.

Rather than acting as a primary initiator of pulmonary fibrosis, dysfunctional adipose tissue may amplify and sustain fibrotic remodeling by shaping the systemic endocrine, inflammatory, and metabolic environment in which lung injury and repair occur. This concept is clinically relevant because fibrotic ILDs frequently coexist with metabolic syndrome, diabetes, cardiovascular disease, gastroesophageal reflux disease, and obstructive sleep apnea, suggesting that pulmonary fibrosis often develops in a host environment already marked by systemic metabolic stress [11].

This review adopts a clinically oriented framework to examine adipose–lung crosstalk in pulmonary fibrosis. First, adipose tissue biology is discussed with emphasis on endocrine and paracrine functions relevant to systemic inflammation and tissue remodeling. Second, epidemiological and imaging evidence linking adiposity to subclinical interstitial lung abnormalities and established fibrotic ILDs are summarized. Third, pathophysiological mechanisms connecting adipose dysfunction to lung fibrosis are explored, integrating adipokine signaling, inflammatory and vascular pathways, epithelial stress responses, fibroblast heterogeneity, cellular senescence, and metabolic reprogramming. Finally, translational implications are discussed, highlighting biomarker development, metabolic stratification, and future mechanism-based therapeutic strategies.

## 2. Adipose Tissue as an Endocrine and Paracrine Organ

Adipose tissue is now recognized as a heterogeneous endocrine and immunometabolic organ whose biological effects depend strongly on depot location, cellular composition, and inflammatory state. In addition to mature adipocytes, it contains a stromal vascular compartment composed of preadipocytes, fibroblasts, endothelial cells, pericytes, and diverse immune-cell populations. Subcutaneous, visceral, perivascular, and ectopic depots differ substantially in structure and immunobiology, with some primarily supporting metabolic homeostasis and others being more closely linked to systemic inflammation and pathological remodeling.

Under physiologic conditions, adipose tissue contributes to systemic homeostasis through balanced secretion of adipokines, cytokines, lipid mediators, and metabolites that regulate insulin sensitivity, immune function, vascular tone, and energy balance [6,7]. By contrast, in obesity and visceral adiposity, adipose tissue undergoes functional remodeling characterized by adipocyte hypertrophy, hypoxia, oxidative and endoplasmic reticulum stress, immune-cell recruitment, and chronic low-grade inflammation [7]. This shift is accompanied by increased leptin, IL-6, TNF-α, and other inflammatory mediators, together with reduced adiponectin and omentin-1, thereby favoring signals associated with maladaptive repair and tissue remodeling [6,7].

These changes make adipose dysfunction relevant not only as a metabolic abnormality but also as a systemic source of inflammatory and profibrotic cues. Similar adipose-associated mechanisms have been implicated in fibrosis of the liver, heart, kidney, and vasculature, supporting the broader concept that dysfunctional adipose tissue can influence tissue repair beyond its anatomical location.

Visceral adipose tissue is more strongly associated with systemic inflammation than subcutaneous fat, while perivascular adipose tissue (PVAT) directly influences vascular tone, endothelial function, immune cell recruitment, and remodeling through local secretion of cytokines and adipokines [12]. Experimental work in vascular injury highlights that PVAT can undergo phenotypic transitions which contribute to inflammatory responses and vascular remodeling, underscoring its role as an active regulator rather than a passive bystander [13]. These processes are clinically relevant to fibrotic ILDs, where pulmonary vascular dysfunction and pulmonary hypertension frequently coexist with interstitial fibrosis and contribute to disease severity and prognosis.

Thus, adipose tissue biology provides a mechanistic basis for examining how systemic metabolic and inflammatory dysfunction may influence fibrotic remodeling in the lung.

## 3. Epidemiology and Imaging: Clinical Studies Linking Adiposity to the Development and Progression of Fibrotic ILD

Clinical studies linking adiposity to fibrotic ILD can be broadly divided into those addressing early fibrotic abnormalities and those examining outcomes in established fibrotic disease. In population-based imaging cohorts, excess visceral and pericardial adiposity is associated with early interstitial abnormalities, whereas in established fibrotic ILD/IPF, BMI- and body composition-based associations with outcome are more heterogeneous and likely influenced by reverse causation. The principal clinical studies are summarized in Table 1.

The strongest evidence linking excess adiposity to early fibrotic lung injury comes from imaging-based population data. In the Multi-Ethnic Study of Atherosclerosis (MESA), greater CT-derived visceral and pericardial adipose tissue was associated with higher high-attenuation areas, increased odds of interstitial lung abnormalities, and lower percent-predicted FVC, supporting a link between metabolically active adipose depots and early fibrotic remodeling [14,15,16]. These findings are particularly informative because they implicate metabolically active adipose depots rather than total body weight alone, thereby supporting the concept that adipose dysfunction may influence early remodeling responses before overt fibrotic disease becomes clinically apparent.

In established fibrotic ILD, several cohorts have reported inverse associations between BMI and mortality. In an early IPF cohort, each 1 kg/m^2^ increase in BMI was associated with lower mortality [17]. Similarly, higher BMI was associated with lower 1-year mortality in fibrotic ILD cohorts, while in a nationwide Korean IPF cohort, underweight status predicted higher mortality and hospitalization, whereas overweight status was associated with lower mortality [18,19]. However, these observations should not be interpreted as evidence that excess adiposity is uniformly protective. In advanced IPF, low BMI may therefore reflect frailty, sarcopenia, systemic catabolism, or more severe disease rather than a causal effect of leanness. Conversely, higher BMI may partly identify patients with greater physiological reserve or earlier-stage disease. This limitation likely explains much of the so-called “obesity paradox” reported in fibrotic lung disease.

Recent body composition studies support the view that imaging-based phenotyping is more informative than BMI alone. In IPF, longitudinal CT-based body composition analysis showed that early decline in body fat after diagnosis was associated with poorer prognosis [20]. In addition, automated 3D CT-based body composition analysis demonstrated prognostic value for survival beyond BMI, including associations of fat-related indices and myosteatosis with outcome [21]. Consistent with the clinical relevance of body habitus in treatment trajectories, lower BMI has also been associated with worse survival and higher risk of nintedanib discontinuation in patients with IPF and progressive fibrotic ILD [22].

Overall, current clinical data supports a link between adiposity/body composition and fibrotic ILD, but they also indicate that the most informative signals arise from imaging-defined adiposity and body composition phenotyping rather than BMI alone, reinforcing the need for more precise metabolic stratification in future ILD cohorts and clinical trials.

**Table 1:** Clinical studies linking adiposity and body composition with early interstitial abnormalities and outcomes in fibrotic ILD/IPF.

Clinical Domain	Study	Population (*n*)	Assessment Method	Endpoint	Main Result
Early abnormalities	Anderson et al. [14]	MESA Cohort	CT visceral/pericardial fat	HAA, ILA, FVC	Visceral adiposity linked to ILA/HAA and lower FVC
Prognosis	Alakhras et al. [17]	IPF (*n* = 197)	BMI	Mortality	Higher BMI linked to lower mortality
Prognosis	Comes et al. [23]	Fibrotic ILD (*n* = 1786)	BMI and weight change	1-year mortality	Higher BMI linked to lower mortality
Prognosis	Yoοn et al. [19]	IPF (*n* = 11,826)	BMI categories	Mortality and hospitalization	Underweight worse outcomes; overweight lower mortality
Body composition	Salhöfer et al. [21]	IPF (*n* = 79)	Automated 3D CT-based body composition	Overall survival	CT body-composition indices predicted survival beyond BMI
Treatment trajectory	Yazaki et al. [22]	IPF (*n* = 212)	BMI	Nintedanib discontinuation and survival	Lower BMI linked to worse survival and discontinuation

## 4. Mechanistic Pathways: How Adipose Dysfunction Conditions the Fibrotic Lung Niche

Adipose dysfunction may condition the fibrotic lung niche through three interconnected mechanisms: endocrine signaling, immune–vascular priming, and modulation of epithelial and fibroblast state transitions. These pathways provide a framework for integrating adipose biology into contemporary models of IPF pathogenesis.

### 4.1. Endocrine Signals Linking Adipose Dysfunction to Fibrotic Remodeling

Adipokines represent one of the most plausible endocrine links between adipose dysfunction and pulmonary fibrosis. By acting on epithelial cells, fibroblasts, macrophages, and vascular cells, adipose-derived mediators may influence whether lung injury resolves through adaptive repair or progresses toward persistent fibrosis [8,24].

#### 4.1.1. Adiponectin: A Protective Adipokine Linked to Fibroblast Plasticity and Repair Restraint

Adiponectin is the most abundant circulating adipokine and is generally associated with insulin sensitization and anti-inflammatory signaling. It exists in multiple isoforms, with high-molecular-weight adiponectin considered the most biologically active, and signals through AdipoR1, AdipoR2, and T-cadherin (CDH13) [25,26,27]. In pulmonary fibrosis models, adiponectin is broadly protective. Mechanistically, CDH13 and p38MAPKγ signaling suppresses matrix stiffness-dependent profibrotic activation of primary human lung fibroblasts and promotes re-establishment of a PPARγ-dependent lipogenic program, thereby counteracting myofibroblast features [28,29]. Consistent with this, CDH13 and p38MAPKγ expression are reduced in IPF lung tissue, suggesting attenuation of an endogenous antifibrotic pathway [28].

Within contemporary concepts of fibroblast heterogeneity and plasticity, adiponectin may help maintain or restore homeostatic fibroblast states rather than acting on a single uniform fibroblast population. Clinically, adiponectin levels in fibrotic ILD appear context-dependent, likely reflecting protective biology, systemic catabolism, and compensatory signaling rather than a simple linear biomarker relationship [8,30,31,32,33,34]. These findings support adiponectin as both a mechanistic mediator and a biomarker candidate, emphasizing the need for cautious interpretation in human disease.

#### 4.1.2. Leptin: A Profibrotic Adipokine Linking Metabolic Inflammation to Fibroblast Persistence

Leptin provides the converse model. It is elevated in obesity, promotes inflammatory activation, suppresses PPARγ-dependent antifibrotic signaling, and enhances myofibroblast differentiation and extracellular matrix production through pathways including PI3K/Akt and mTOR [35,36,37]. It may also act upstream of fibroblast activation by influencing epithelial stress responses and maladaptive repair. Although leptin has not been established as a direct driver of RUNX2-associated epithelial transitions, leptin-rich and metabolically inflamed environments may lower the threshold for maladaptive epithelial–stromal reprogramming by reinforcing inflammatory and profibrotic signaling [38].

Clinically, leptin is elevated during acute exacerbations of IPF and mediates part of the association between visceral adiposity and early interstitial abnormalities in population cohorts [15,33,39]. Taken together, adiponectin and leptin illustrate how adipose dysfunction can shift the systemic endocrine milieu away from homeostatic restraint toward profibrotic persistence.

#### 4.1.3. Omentin-1, Resistin, Chemerin, and Related Mediators

Other adipose-derived mediators further support a balance between pro-resolving and profibrotic signals. Omentin-1, a visceral adipose-derived adipokine, has emerged as a potential mediator of fibrosis resolution. In experimental models, omentin-1 deficiency worsens fibrosis, whereas exogenous omentin-1 accelerates regression of established fibrosis, partly by shifting activated fibroblasts away from a contractile state and toward a more lipogenic and homeostatic phenotype through inhibition of YAP-dependent mechanotransduction and reactivation of PPARγ-linked programs [40]. This positions omentin-1 as a candidate pro-resolving signal within the adipose–lung axis.

By contrast, resistin and chemerin appear to function primarily as inflammatory amplifiers. Resistin has been linked to fibrotic lung involvement, particularly in systemic sclerosis-associated ILD, and may promote chronic inflammation and myofibroblast persistence [41,42,43]. Chemerin, which signals through CMKLR1, is elevated in several inflammatory conditions and has been reported at higher levels in IPF and sarcoidosis, where it may facilitate macrophage recruitment, M1 polarization, and vascular inflammation [8,44,45,46,47]. CTRP9, another adipocyte-derived mediator, has also been associated with ILD severity in SSc-ILD [48]. Although these molecules are less well characterized than adiponectin and leptin, together they reinforce the concept that adipose dysfunction generates a systemic endocrine environment that can either restrain or amplify fibrotic remodeling (Table 2).

### 4.2. Immune and Vascular Niche Priming in the Injured Lung: PVAT, Inflammasomes, Macrophages, and Senescence-Associated Signaling

Dysfunctional adipose tissue generates a chronic inflammatory background that may prime the injured lung niche. In obesity and metabolic dysfunction, visceral and perivascular adipose depots become enriched with activated macrophages and inflammasome signaling, leading to sustained production of IL-1β, IL-6, TNF-α, and related mediators [7,14,24]. Although these signals are unlikely to act as direct pulmonary effectors, they may lower the threshold for exaggerated injury responses and maladaptive repair.

PVAT is particularly relevant because it functions as a dynamic immunometabolic compartment that regulates vascular tone, endothelial activation, leukocyte recruitment, and remodeling rather than serving as passive structural fat [13]. This has clinical relevance in fibrotic ILD because pulmonary vascular dysfunction and pulmonary hypertension frequently coexist with interstitial fibrosis and contribute to exercise limitation and worse prognosis.

Recent single-cell studies provide additional mechanistic precision. Coronary PVAT from advanced vascular disease contains expanded SPP1-positive macrophage populations associated with stromal fibrosis [49]. Similar SPP1-expressing macrophages have been identified in fibrotic lung tissue, where they are localized to remodeling niches and support fibroblast activation and matrix deposition [50]. These observations do not imply direct migration of adipose macrophages into the lung; rather, they suggest convergence between chronic adipose inflammation and injured lung tissue on shared profibrotic immune programs. In this context, macrophage–stromal crosstalk may represent one route through which systemic immunometabolic dysfunction reinforces local fibrosis.

### 4.3. Epithelial Maladaptation, Transitional States, and Senescence-Associated Persistence

To integrate the adipose–lung axis into contemporary IPF biology, it must be considered alongside epithelial-centered models of pathogenesis. Current fibrosis frameworks place repeated alveolar epithelial injury and maladaptive epithelial repair at the core of disease initiation. Single-cell and lineage-resolved studies have shown that epithelial repair in fibrosis involves persistent transitional epithelial states, including KRT8-positive or PATS-like (pre-alveolar type-1 transitional cell state) intermediates and aberrant basaloid programs, that may become maladaptive when repair is incomplete or chronically stressed [51,52].

Within this framework, dysfunctional adipose tissue need not directly injure the epithelium to be biologically relevant. Instead, adipose-derived inflammatory and metabolic signals may create a systemic environment in which epithelial stress responses are more likely to persist, transitional programs fail to resolve, and epithelial–stromal crosstalk becomes self-sustaining. EMT-like or epithelial–mesenchymal transitional processes remain debated as direct sources of fibroblasts but are increasingly recognized as markers of maladaptive epithelial plasticity and profibrotic signaling in IPF [53].

This integrative model also intersects with cellular senescence. Senescent epithelial cells, fibroblasts, and immune cells contribute to fibrosis persistence through the senescence-associated secretory phenotype (SASP), which is enriched in IL-6, IL-1β, chemokines, and matrix-remodeling signals [54]. Transitional epithelial states themselves have been linked to senescence-associated programs in pulmonary fibrosis, suggesting that failed repair and senescence are mechanistically intertwined. Although direct evidence in fibrotic ILD remains limited, chronic adipose inflammation could plausibly intensify this SASP-rich environment by sustaining inflammatory cues that favor persistence of senescent cell programs.

Although not adipokines, FGF-related pathways provide an additional example of how systemic metabolic mediators may influence the fibrotic niche. FGF21, FGF23/FGFR4, FGF9, and FGF18 have been implicated in tissue repair, inflammation, and fibroblast activity in experimental lung injury and pulmonary fibrosis [55,56,57,58].

### 4.4. Fibroblast Heterogeneity, Epithelial Plasticity, and Metabolic Reprogramming as Downstream Effectors

The downstream consequences of endocrine, inflammatory, and epithelial inputs are best understood at the level of fibroblast state remodeling. Contemporary single-cell transcriptomic studies have shown that fibroblasts in fibrotic lung disease are heterogeneous and dynamic rather than a single activated population. Human pulmonary fibrosis contains multiple stromal states, including matrix-producing, inflammatory, and transitional mesenchymal populations, indicating that fibrosis persistence depends on stabilization of specific pathogenic states rather than uniform fibroblast activation [59,60].

Within this context, metabolic reprogramming is a core fibrotic mechanism. Experimental studies have shown that fibroblasts can transition between lipogenic and myogenic phenotypes during fibrosis progression and resolution and that inflammatory intermediate states shaped by IL-1β and IL-17A can stabilize profibrotic trajectories [61,62,63]. More broadly, current work in pulmonary fibrosis identifies altered glucose, lipid, mitochondrial, and amino acid metabolism as central regulators of fibroblast persistence and matrix production [64].

Adipose biology is particularly relevant here because adipose dysfunction alters circulating adiponectin, leptin, inflammatory cytokines, and lipid mediators, all of which may influence pathways such as PPARγ that govern lipogenic identity and oppose TGF-β-driven profibrotic activation [24,28,35]. Thus, adipose-derived signals are unlikely to switch fibrosis “on” or “off”; rather, they may bias epithelial, immune, and fibroblast states toward persistence rather than resolution.

**Figure 1:**
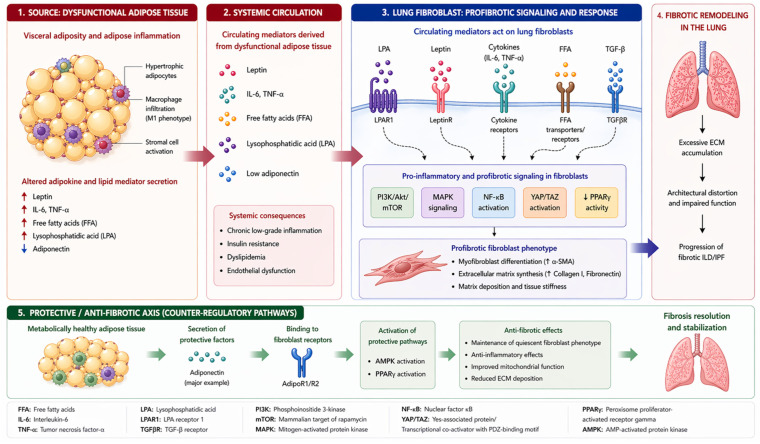
Proposed model of systemic adipose–lung crosstalk in pulmonary fibrosis.

The concept of systemic adipose–lung crosstalk in pulmonary fibrosis is summarized in Figure 1.

## 5. Clinical Modifiers and Comorbidities: Why Adipose Dysfunction Matters at the Bedside

The clinical relevance of adipose dysfunction is likely to be most apparent through the comorbidity burden that commonly accompanies fibrotic ILD. Patients with IPF frequently present conditions biologically linked to adipose dysfunction, including gastroesophageal reflux disease, obstructive sleep apnea, cardiovascular disease, obesity, insulin resistance, and diabetes. These disorders may promote recurrent epithelial stress, intermittent hypoxia, vascular dysfunction, oxidative stress, and chronic low-grade inflammation, thereby creating a systemic environment that can amplify fibrosis progression.

Contemporary clinical reviews emphasize that comorbidities in IPF are not merely coincidental findings but important determinants of symptoms, quality of life, treatment tolerance, hospitalization risk, and survival [11]. Their clustering within the broader metabolic syndrome spectrum further supports the relevance of adipose biology in day-to-day disease behavior. These observations support more systematic assessment of metabolic and adipose-related comorbidities in fibrotic ILD, particularly when evaluating disease burden, prognosis, and treatment tolerance.

## 6. Therapeutic Implications of the Adipose–Lung Axis

The therapeutic relevance of the adipose–lung axis lies primarily in biological stratification and phenotype-directed care rather than immediate introduction of adipose-targeted drugs. Three translational directions are most relevant: targeting metabolic reprogramming pathways, optimizing metabolic health as part of supportive care, and exploring adipokine-related interventions as longer-term investigational strategies.

### 6.1. Metabolic Reprogramming as the Most Developed Therapeutic Concept

Among the pathways discussed in this review, metabolic reprogramming currently provides the strongest mechanistic basis for therapeutic translation. Experimental studies indicate that fibrosis persistence is linked to stable profibrotic fibroblast states, whereas restoration of lipogenic programs may favor resolution. Pathways such as PPARγ are therefore particularly relevant because they connect adipose biology with fibroblast plasticity [65]. In experimental models, PPARγ agonists such as rosiglitazone and pioglitazone suppress collagen and α-SMA induction in TGF-β-stimulated lung fibroblasts and attenuate fibrosis in bleomycin-injured mice [61,66].

Similarly, metformin promotes myofibroblast-to-lipogenic reprogramming through AMPK- and PPARγ-dependent pathways and facilitates fibrosis resolution in preclinical models [67,68]. More recent work also suggests that combined PPARγ and PPAR-β/δ activation may enhance antifibrotic effects [69].

These findings are consistent with current concepts of fibroblast plasticity and metabolic control of fibrosis. Human evidence, however, remains limited. Observational studies of metformin in IPF have yielded mixed results, and clinical data on PPARγ-directed strategies in fibrotic ILD are lacking [70,71,72]. At present, this approach should therefore be viewed as a mechanistically prioritized research direction rather than an established therapeutic option.

### 6.2. Clinical Relevance Already Exists at the Level of Metabolic Phenotype

Common metabolic comorbidities discussed above may represent modifiable contributors to disease burden and treatment tolerance. In practical terms, weight management, structured exercise, pulmonary rehabilitation, and optimized treatment of metabolic comorbidities may not reverse fibrosis directly but may reduce systemic inflammatory burden, improve physiological reserve, and support antifibrotic treatment persistence [11,73,74]. This does not yet justify disease-specific metabolic interventions in all patients, but it supports considering metabolic phenotype in routine assessment and future trial design.

### 6.3. Adipokine-Targeted Interventions Remain Exploratory

Direct modulation of adipokine signaling remains more speculative. Restoring adiponectin-related pathways or inhibiting profibrotic mediators such as leptin is mechanistically appealing because these signals lie upstream of epithelial stress, immune–stromal crosstalk, and fibroblast persistence [24,34,75,76,77,78]. However, these approaches have not been tested in dedicated pulmonary fibrosis trials, and their systemic physiological roles raise questions about specificity and tolerability.

Adipokine-directed strategies are therefore best regarded as exploratory extensions of the adipose–lung hypothesis rather than near-term clinical candidates. Their current value is conceptual: they help define which systemic signals might eventually be targeted if metabolically enriched patient subsets can first be identified.

Overall, the near-term therapeutic relevance of the adipose–lung axis lies in biomarker-guided and phenotype-directed studies rather than immediate adipose-targeted intervention.

## 7. Clinical Translation and Potential Biomarkers

Clinical translation of the adipose–lung axis will require biomarkers that capture metabolic state, inflammatory tone, and body composition. These tools may support risk stratification, biological phenotyping, and treatment-response assessment.

Population data indicate that adipokines such as leptin and inflammatory mediators such as IL-6 associate with subclinical ILD features and link visceral adiposity to imaging abnormalities [14]. In parallel, genetic causal inference supports adiponectin as a protective factor for ILD risk, which strengthens the rationale for incorporating adiponectin-related signatures into prospective cohorts and trials [79]. Clinical studies in fibrotic ILD further suggest that adiponectin level and oligomerization patterns may relate to disease presence and functional status [34].

Beyond adipokines, circulating lipid and lipoprotein-related biomarkers may capture downstream metabolic consequences of adipose dysfunction and provide complementary prognostic information in IPF. Lipoprotein-related markers, bioactive lipid mediators, and serum lipidomic signatures have recently emerged as candidate biomarkers associated with pulmonary function, disease progression, survival, and acute exacerbation risk. Representative clinical studies are summarized in Table 3.

Building on the prognostic imaging data discussed above, CT-based body composition analysis could serve as a translational biomarker platform by quantifying visceral adiposity, thoracic fat depots, and sarcopenic or cachectic phenotypes [21]. The broader cardiovascular literature emphasizes PVAT imaging and fat attenuation approaches as potential markers of adipose inflammation, concepts that may be adapted to thoracic imaging in fibrotic ILD [12]. Together, these tools could support integrative “metabolo-fibrotic” endotyping that combines body composition, adipokine profiles, lipid biomarkers, metabolic syndrome features, and lung-specific disease metrics.

From a therapeutic perspective, biomarker-guided patient selection may be essential. Patients with metabolically dysregulated phenotypes, including visceral adiposity, insulin resistance, sarcopenic obesity, or pro-inflammatory adipokine profiles, may derive greater benefit from metabolic or adipose-related interventions than unselected fibrotic ILD populations. Conversely, advanced cachexia or severe systemic frailty may influence treatment tolerance and should be considered in trial design. These biomarker strategies may help select metabolically enriched patient subsets and evaluate whether interventions modify adipose–lung crosstalk alongside clinical outcomes.

## 8. Future Directions

Future research should move from association toward causality. A key priority is to determine whether adipose dysfunction precedes early fibrotic abnormalities, accelerates progression after disease onset, or primarily reflects advanced disease and systemic catabolism. Longitudinal ILD cohorts integrating body-composition imaging, adipokine profiling, lipid biomarkers, and metabolic phenotyping will be essential to address this question.

A second priority is to test whether metabolic stratification improves clinical trial design. If adipose-related pathways are most relevant in metabolically dysregulated phenotypes, unselected ILD populations may dilute potential treatment effects. Biomarker-enriched and phenotype-directed studies will therefore be needed to evaluate metabolic or adipose-related interventions more rigorously.

Mechanistically, future work should define how systemic metabolic dysfunction interacts with epithelial repair failure, senescence-associated signaling, immune–stromal crosstalk, and fibroblast state transitions in the human lung. Combining single-cell transcriptomics, functional metabolic studies, and human tissue validation will be critical.

Ultimately, the value of the adipose–lung model will depend on whether it can improve biological stratification and guide mechanism-based interventions in fibrotic ILD.

## 9. Concluding Remarks

Pulmonary fibrosis remains a lung-centered disease driven by epithelial injury, abnormal repair, and persistent fibroblast activation. The evidence reviewed here suggests that systemic metabolic factors, particularly adipose dysfunction, may modify disease progression through inflammatory, vascular, endocrine, and metabolic pathways.

This adipose–lung perspective integrates epidemiologic, imaging, biomarker, and experimental data without replacing epithelial-centered models of IPF pathogenesis. Instead, it adds a systemic layer that may help explain heterogeneity in disease behavior, comorbidity burden, and treatment tolerance.

At present, its main clinical value lies in improved phenotyping and patient stratification rather than immediate adipose-targeted therapy. Integrating body-composition analysis, adipokine and lipid biomarkers, and metabolic profiling with lung-specific disease metrics may support more precise management of fibrotic ILD.

## Figures and Tables

**Table 2:** Major Adipokines involved in pulmonary fibrosis.

Adipokine	Role in Fibrosis	Key Pathways	Findings in ILD
Adiponectin (HMW)	Antifibrotic, Anti-inflammatory	AMPK/PPARα, T-cadherin-p38MAPKγ, NF-KB	Context-dependent; altered levels/oligomerization associated with lung function
Leptin	Profibrotic, Pro-inflammatory	TGFβ, PI3K/AKT, JAK/STAT, Th17 activation, M1 macrophages recruitment	Increased in advanced IPF and AE-ILDs
Omentin-1	Anti-fibrotic, pro-resolving	AMPK/PPARγ, reduction in PKM2-YAP	Limited human data; pro-resolving effects in models
Resistin	Profibrotic, Pro-inflammatory	NF-KB, IL-6/IL-1β/TNFa	Increased in SSc-ILD and CTD-ILD
Chemerin	Pro-inflammatory, Vascular remodeling	CMKLR1 chemotaxis, endothelial activation	Increased in IPF and sarcoidosis. Potential role in pulmonary hypertension via PVAT inflammation
CTRP9	Anti-inflammatory, vasculoprotective	AMPK activation, anti-oxidative stress	Higher levels linked to worse lung function

**Table 3:** Representative clinical studies evaluating circulating lipid and lipid-related biomarkers in IPF. Current evidence suggests potential prognostic or disease-monitoring value, although several candidates require further validation. Abbreviations: ApoA-I, apolipoprotein A-I; DLco, diffusing capacity for carbon monoxide; GAP, gender–age–physiology index; HDL, high-density lipoprotein; IPF, idiopathic pulmonary fibrosis; LC-MS, liquid chromatography-mass spectrometry; LPA, lysophosphatidic acid; NMR, nuclear magnetic resonance; PEA, palmitoyl ethanolamide.

Study	Biomarker	Sample	Population	Clinical Association	Main Finding
Oh et al., 2024 [80]	ApoA-I	Serum	IPF (*n* = 371)	Mortality; disease severity	Lower ApoA-I predicted mortality and correlated with lower DLco/higher GAP
Barochia et al., 2021 [81]	Small HDL particles	Serum/NMR lipoprotein profiling	IPF (*n* = 59)	Death or lung transplantation	Higher small HDL associated with lower risk of death/transplantation
Neighbors et al., 2023 [54]	LPA species	Plasma	IPF (*n* = 173)	Disease progression	Specific LPA species associated with IPF progression
Cai et al., 2025 [82]	PEA and 2-amino-1,3,4-octadecanetriol	Serum/LC–MS metabolomics	IPF (*n* = 30)	Diagnosis; severity; prognosis	Lipid metabolites showed preliminary diagnostic/prognostic associations

## Data Availability

No new data were created or analyzed in this study.

## References

[1] Distler J.H.W., Györfi A.H., Ramanujam M., Whitfield M.L., Königshoff M., Lafyatis R. (2019). Shared and distinct mechanisms of fibrosis. Nat. Rev. Rheumatol..

[2] Althobiani M.A., Russell A.M., Jacob J., Ranjan Y., Folarin A.A., Hurst J.R., Porter J.C. (2024). Interstitial lung disease: A review of classification, etiology, epidemiology, clinical diagnosis, pharmacological and non-pharmacological treatment. Front. Med..

[3] Maher T.M., Lancaster L.H., Jouneau S., Morrison L., Lederer D.J., Molina-Molina M., Bendstrup E., Kirchgaessler K.U., Gilberg F., Axmann J. (2019). Pirfenidone Treatment in Individuals with Idiopathic Pulmonary Fibrosis: Impact of Timing of Treatment Initiation. Ann. Am. Thorac. Soc..

[4] Lancaster L., Crestani B., Hernandez P., Inoue Y., Wachtlin D., Loaiza L., Quaresma M., Stowasser S., Richeldi L. (2019). Safety and survival data in patients with idiopathic pulmonary fibrosis treated with nintedanib: Pooled data from six clinical trials. BMJ Open Respir. Res..

[5] Richeldi L., Azuma A., Cottin V., Kreuter M., Maher T.M., Martinez F.J., Oldham J.M., Valenzuela C., Clerisme-Beaty E., Gordat M. (2025). Nerandomilast in Patients with Idiopathic Pulmonary Fibrosis. N. Engl. J. Med..

[6] El Husseini K., Poté N., Jaillet M., Mordant P., Mal H., Frija-Masson J., Borie R., Cazes A., Crestani B., Mailleux A. (2023). Adipocytes, adipokines and metabolic alterations in pulmonary fibrosis. Rev. Mal. Respir..

[7] Kawai T., Autieri M.V., Scalia R. (2021). Adipose tissue inflammation and metabolic dysfunction in obesity. Am. J. Physiol. Cell Physiol..

[8] Zielinski M., Chwalba A., Jastrzebski D., Ziora D. (2023). Adipokines in interstitial lung diseases. Respir. Physiol. Neurobiol..

[9] Li M., McKeon B.A., Frid M., Stenmark K.R. (2025). The Role of Perivascular Adipose Tissue Derived Complement Factors in Regulating Inflammation and Vascular Remodeling of Large Pulmonary Arteries in Pulmonary Hypertension. Am. J. Respir. Crit. Care Med..

[10] Ruiz-Villalba A., Pardo-Saganta A. (2025). Emerging concepts and novel mechanisms in organ fibrosis. npj Regen. Med..

[11] Salotti A., Chianese M., Romallo A., De Nes A., Angoni D., Galantino A., Chernovsky M., Mondini L., Salton F., Confalonieri P. (2025). Comorbidities’ Effect on IPF: Pathogenesis and Management. Biomedicines.

[12] Antoniades C., Tousoulis D., Vavlukis M., Fleming I., Duncker D.J., Eringa E., Manfrini O., Antonopoulos A.S., Oikonomou E., Padró T. (2023). Perivascular adipose tissue as a source of therapeutic targets and clinical biomarkers. Eur. Heart J..

[13] Adachi Y., Ueda K., Nomura S., Ito K., Katoh M., Katagiri M., Yamada S., Hashimoto M., Zhai B., Numata G. (2022). Beiging of perivascular adipose tissue regulates its inflammation and vascular remodeling. Nat. Commun..

[14] Anderson M.R., Kim J.S., Allison M., Giles J.T., Hoffman E.A., Ding J., Barr R.G., Podolanczuk A. (2021). Adiposity and Interstitial Lung Abnormalities in Community-Dwelling Adults: The MESA Cohort Study. Chest.

[15] Kim J.S., Anderson M.R., Podolanczuk A.J., Kawut S.M., Allison M.A., Raghu G., Hinckley-Stuckovsky K., Hoffman E.A., Tracy R.P., Barr R.G. (2020). Associations of Serum Adipokines With Subclinical Interstitial Lung Disease Among Community-Dwelling Adults: The Multi-Ethnic Study of Atherosclerosis (MESA). Chest.

[16] Anderson M.R., Kim J.S., Podolanczuk A., Ding J., Al-Naamani N., Allison M., Christie J., Diamond J. (2024). Nonlinear associations between computed tomography-measures of adiposity and long pentraxin-3 in the Multi-Ethnic Study of Atherosclerosis. Obes. Sci. Pract..

[17] Alakhras M., Decker P.A., Nadrous H.F., Collazo-Clavell M., Ryu J.H. (2007). Body mass index and mortality in patients with idiopathic pulmonary fibrosis. Chest.

[18] Nam Y., Yoon E.C., Yoon H.-Y. (2026). Association between body mass index and prognosis in interstitial lung disease: Systematic review and meta-analysis. Front. Med..

[19] Yoon H.-Y., Kim H., Bae Y., Song J.W. (2024). Body mass index is associated with clinical outcomes in idiopathic pulmonary fibrosis. Sci. Rep..

[20] Lee J.Y., Yoon S.H., Goo J.M., Park J., Lee J.H. (2024). Association between body fat decrease during the first year after diagnosis and the prognosis of idiopathic pulmonary fibrosis: CT-based body composition analysis. Respir. Res..

[21] Salhöfer L., Bonella F., Meetschen M., Umutlu L., Forsting M., Schaarschmidt B.M., Opitz M., Beck N., Zensen S., Hosch R. (2024). CT-based body composition analysis and pulmonary fat attenuation volume as biomarkers to predict overall survival in patients with non-specific interstitial pneumonia. Eur. Radiol. Exp..

[22] Yazaki K., Matsuyama M., Satoh H., Miyazaki K., Arai N., Ishii Y., Endo T., Kaburagi T., Kawakami T., Iijima H. (2025). Gender-age-physiology stage and body mass index are useful predictors of nintedanib discontinuation and prognosis in patients with idiopathic pulmonary fibrosis and progressive fibrotic interstitial lung diseases. BMC Pulm. Med..

[23] Comes A., Wong A.W., Fisher J.H., Morisset J., Johannson K.A., Farrand E., Fell C.D., Kolb M., Manganas H., Cox G. (2022). Association of BMI and Change in Weight With Mortality in Patients With Fibrotic Interstitial Lung Disease. Chest.

[24] Macklin M., Thompson C., Kawano-Dourado L., Bauer Ventura I., Weschenfelder C., Trostchansky A., Marcadenti A., Tighe R.M. (2023). Linking Adiposity to Interstitial Lung Disease: The Role of the Dysfunctional Adipocyte and Inflammation. Cells.

[25] Fang H., Judd R.L. (2018). Adiponectin Regulation and Function. Compr. Physiol..

[26] Hug C., Wang J., Ahmad N.S., Bogan J.S., Tsao T.S., Lodish H.F. (2004). T-cadherin is a receptor for hexameric and high-molecular-weight forms of Acrp30/adiponectin. Proc. Natl. Acad. Sci. USA.

[27] Yamauchi T., Kamon J., Ito Y., Tsuchida A., Yokomizo T., Kita S., Sugiyama T., Miyagishi M., Hara K., Tsunoda M. (2003). Cloning of adiponectin receptors that mediate antidiabetic metabolic effects. Nature.

[28] Nemeth J., Skronska-Wasek W., Keppler S., Schundner A., Groß A., Schoenberger T., Quast K., El Kasmi K.C., Ruppert C., Günther A. (2024). Adiponectin suppresses stiffness-dependent, profibrotic activation of lung fibroblasts. Am. J. Physiol. Lung Cell. Mol. Physiol..

[29] Nemeth J., Schundner A., Quast K., Winkelmann V.E., Frick M. (2020). A Novel Fibroblast Reporter Cell Line for in vitro Studies of Pulmonary Fibrosis. Front. Physiol..

[30] Chung C.P., Long A.G., Solus J.F., Rho Y.H., Oeser A., Raggi P., Stein C.M. (2009). Adipocytokines in systemic lupus erythematosus: Relationship to inflammation, insulin resistance and coronary atherosclerosis. Lupus.

[31] Sada K.E., Yamasaki Y., Maruyama M., Sugiyama H., Yamamura M., Maeshima Y., Makino H. (2006). Altered levels of adipocytokines in association with insulin resistance in patients with systemic lupus erythematosus. J. Rheumatol..

[32] Toussirot E., Gaugler B., Bouhaddi M., Nguyen N.U., Saas P., Dumoulin G. (2010). Elevated adiponectin serum levels in women with systemic autoimmune diseases. Mediat. Inflamm..

[33] Enomoto N., Oyama Y., Yasui H., Karayama M., Hozumi H., Suzuki Y., Kono M., Furuhashi K., Fujisawa T., Inui N. (2019). Analysis of serum adiponectin and leptin in patients with acute exacerbation of idiopathic pulmonary fibrosis. Sci. Rep..

[34] Nigro E., D’Agnano V., Pagliaro R., Mallardo M., Bianco A., Picone C., D’Errico A.G., Daniele A., Perrotta F. (2025). Exploring the role of serum adiponectin and its holigomerization in fibrotic interstitial lung diseases: Results from a cross-sectional study. BMC Pulm. Med..

[35] Jain M., Budinger G.R., Lo A., Urich D., Rivera S.E., Ghosh A.K., Gonzalez A., Chiarella S.E., Marks K., Donnelly H.K. (2011). Leptin promotes fibroproliferative acute respiratory distress syndrome by inhibiting peroxisome proliferator-activated receptor-γ. Am. J. Respir. Crit. Care Med..

[36] Chen B., Lyu Q., Zhang M., Zhang M., Liu W., Zhu S. (2015). [Leptin induces human lung fibroblast to trans-differentiate into myofibroblast]. Xi Bao Yu Fen Zi Mian Yi Xue Za Zhi.

[37] Gui X., Chen H., Cai H., Sun L., Gu L. (2018). Leptin promotes pulmonary fibrosis development by inhibiting autophagy via PI3K/Akt/mTOR pathway. Biochem. Biophys. Res. Commun..

[38] Fang Y., Chung S.S.W., Xu L., Xue C., Liu X., Jiang D., Li R., Korogi Y., Yuan K., Saqi A. (2025). RUNX2 promotes fibrosis via an alveolar-to-pathological fibroblast transition. Nature.

[39] Cao M., Swigris J.J., Wang X., Cao M., Qiu Y., Huang M., Xiao Y., Cai H. (2016). Plasma Leptin Is Elevated in Acute Exacerbation of Idiopathic Pulmonary Fibrosis. Mediat. Inflamm..

[40] Zhang Y., Fu J., Li C., Chang Y., Li X., Cheng H., Qiu Y., Shao M., Han Y., Feng D. (2023). Omentin-1 induces mechanically activated fibroblasts lipogenic differentiation through pkm2/yap/pparγ pathway to promote lung fibrosis resolution. Cell. Mol. Life Sci..

[41] Nieva-Vazquez A., Pérez-Fuentes R., Torres-Rasgado E., López-López J.G., Romero J.R. (2014). Serum resistin levels are associated with adiposity and insulin sensitivity in obese Hispanic subjects. Metab. Syndr. Relat. Disord..

[42] Sawicka K., Michalska-Jakubus M., Kowal M., Potembska E., Krasowska D. (2017). Resistin: A possible biomarker of organ involvement in systemic sclerosis patients?. Clin. Exp. Rheumatol..

[43] Lin Q., Johns R.A. (2020). Resistin family proteins in pulmonary diseases. Am. J. Physiol. Lung Cell. Mol. Physiol..

[44] Zou R., Wang M.H., Chen Y., Fan X., Yang B., Du J., Wang X.B., Liu K.X., Zhou J. (2019). Hydrogen-Rich Saline Attenuates Acute Lung Injury Induced by Limb Ischemia/Reperfusion via Down-Regulating Chemerin and NLRP3 in Rats. Shock.

[45] Boyuk B., Guzel E.C., Atalay H., Guzel S., Mutlu L.C., Kucukyalçin V. (2015). Relationship between plasma chemerin levels and disease severity in COPD patients. Clin. Respir. J..

[46] Li J., Lu Y., Li N., Li P., Wang Z., Ting W., Liu X., Wu W. (2020). Chemerin: A Potential Regulator of Inflammation and Metabolism for Chronic Obstructive Pulmonary Disease and Pulmonary Rehabilitation. Biomed. Res. Int..

[47] Grygiel-Górniak B., Grzelak T., Czyżewska K., Puszczewicz M. (2018). Chemerin, Resistin, and Adiponectin in Patients with Connective Tissue Diseases. J. Med. Biochem..

[48] Yang M.M., Balmert L.C., Marangoni R.G., Carns M., Hinchcliff M., Korman B.D., Varga J. (2023). Circulating CTRP9 Is Associated With Severity of Systemic Sclerosis–Associated Interstitial Lung Disease. Arthritis Care Res..

[49] Fu M., Shu S., Peng Z., Liu X., Chen X., Zeng Z., Yang Y., Cui H., Zhao R., Wang X. (2023). Single-Cell RNA Sequencing of Coronary Perivascular Adipose Tissue From End-Stage Heart Failure Patients Identifies SPP1(+) Macrophage Subpopulation as a Target for Alleviating Fibrosis. Arterioscler. Thromb. Vasc. Biol..

[50] Adams T.S., Schupp J.C., Poli S., Ayaub E.A., Neumark N., Ahangari F., Chu S.G., Raby B.A., DeIuliis G., Januszyk M. (2020). Single-cell RNA-seq reveals ectopic and aberrant lung-resident cell populations in idiopathic pulmonary fibrosis. Sci. Adv..

[51] Kobayashi Y., Tata A., Konkimalla A., Katsura H., Lee R.F., Ou J., Banovich N.E., Kropski J.A., Tata P.R. (2020). Persistence of a regeneration-associated, transitional alveolar epithelial cell state in pulmonary fibrosis. Nat. Cell Biol..

[52] Strunz M., Simon L.M., Ansari M., Mattner L.F., Angelidis I., Mayr C.H., Kathiriya J., Yee M., Ogar P., Sengupta A. (2019). Longitudinal single cell transcriptomics reveals Krt8+ alveolar epithelial progenitors in lung regeneration. bioRxiv.

[53] Li W., Xie Y., Chen Z., Cao D., Wang Y. (2025). Epithelial-mesenchymal transition in pulmonary fibrosis: Molecular mechanisms and emerging therapeutic strategies. Front. Med..

[54] Neighbors M., Li Q., Zhu S.J., Liu J., Wong W.R., Jia G., Sandoval W., Tew G.W. (2023). Bioactive lipid lysophosphatidic acid species are associated with disease progression in idiopathic pulmonary fibrosis. J. Lipid. Res..

[55] Ghanem M., Archer G., Justet A., Jaillet M., Vasarmidi E., Mordant P., Castier Y., Mal H., Cazes A., Poté N. (2025). FGF21 Signaling Exerts Antifibrotic Properties during Pulmonary Fibrosis. Am. J. Respir. Crit. Care Med..

[56] Ghanem M., Justet A., Jaillet M., Vasarmidi E., Boghanim T., Hachem M., Vadel A., Joannes A., Mordant P., Balayev A. (2024). Identification of FGFR4 as a regulator of myofibroblast differentiation in pulmonary fibrosis. Am. J. Physiol. Lung Cell. Mol. Physiol..

[57] Justet A., Ghanem M., Boghanim T., Hachem M., Vasarmidi E., Jaillet M., Vadel A., Joannes A., Mordant P., Bonniaud P. (2022). FGF19 Is Downregulated in Idiopathic Pulmonary Fibrosis and Inhibits Lung Fibrosis in Mice. Am. J. Respir. Cell Mol. Biol..

[58] Joannes A., Brayer S., Besnard V., Marchal-Sommé J., Jaillet M., Mordant P., Mal H., Borie R., Crestani B., Mailleux A.A. (2016). FGF9 and FGF18 in idiopathic pulmonary fibrosis promote survival and migration and inhibit myofibroblast differentiation of human lung fibroblasts in vitro. Am. J. Physiol. Lung Cell. Mol. Physiol..

[59] Wang J., Zhang L., Luo L., He P., Xiong A., Jiang M., Liu Y., Liu S., Ran Q., Wu D. (2022). Characterizing cellular heterogeneity in fibrotic hypersensitivity pneumonitis by single-cell transcriptional analysis. Cell Death Discov..

[60] Habermann A.C., Gutierrez A.J., Bui L.T., Yahn S.L., Winters N.I., Calvi C.L., Peter L., Chung M.I., Taylor C.J., Jetter C. (2020). Single-cell RNA sequencing reveals profibrotic roles of distinct epithelial and mesenchymal lineages in pulmonary fibrosis. Sci. Adv..

[61] El Agha E., Moiseenko A., Kheirollahi V., De Langhe S., Crnkovic S., Kwapiszewska G., Szibor M., Kosanovic D., Schwind F., Schermuly R.T. (2017). Two-Way Conversion between Lipogenic and Myogenic Fibroblastic Phenotypes Marks the Progression and Resolution of Lung Fibrosis. Cell Stem Cell.

[62] Panagiotidis G.D., Vasquez-Pacheco E., Chu X., Seeger W., El Agha E., Bellusci S., Lingampally A. (2025). Revisiting pulmonary fibrosis: Inflammatory dynamics of the lipofibroblast-to-inflammatory lipofibroblast-to-activated myofibroblast reversible switch. Front. Immunol..

[63] Zheng H., Zhang L., Wang C., Wang Y., Zeng C. (2025). Metabolic dysregulation in pulmonary fibrosis: Insights into amino acid contributions and therapeutic potential. Cell Death Discov..

[64] Li J., Zhai X., Sun X., Cao S., Yuan Q., Wang J. (2022). Metabolic reprogramming of pulmonary fibrosis. Front. Pharmacol..

[65] Rehan V.K., Torday J.S. (2012). PPARγ Signaling Mediates the Evolution, Development, Homeostasis, and Repair of the Lung. PPAR Res..

[66] Genovese T., Cuzzocrea S., Di Paola R., Mazzon E., Mastruzzo C., Catalano P., Sortino M., Crimi N., Caputi A.P., Thiemermann C. (2005). Effect of rosiglitazone and 15-deoxy-Delta12,14-prostaglandin J2 on bleomycin-induced lung injury. Eur. Respir. J..

[67] Kheirollahi V., Wasnick R.M., Biasin V., Vazquez-Armendariz A.I., Chu X., Moiseenko A., Weiss A., Wilhelm J., Zhang J.-S., Kwapiszewska G. (2019). Metformin induces lipogenic differentiation in myofibroblasts to reverse lung fibrosis. Nat. Commun..

[68] Rangarajan S., Bone N.B., Zmijewska A.A., Jiang S., Park D.W., Bernard K., Locy M.L., Ravi S., Deshane J., Mannon R.B. (2018). Metformin reverses established lung fibrosis in a bleomycin model. Nat. Med..

[69] Boateng E., Bonilla-Martinez R., Ahlemeyer B., Garikapati V., Alam M.R., Trompak O., Oruqaj G., El-Merhie N., Seimetz M., Ruppert C. (2024). It takes two peroxisome proliferator-activated receptors (PPAR-β/δ and PPAR-γ) to tango idiopathic pulmonary fibrosis. Respir. Res..

[70] Teague T.T., Payne S.R., Kelly B.T., Dempsey T.M., McCoy R.G., Sangaralingham L.R., Limper A.H. (2022). Evaluation for clinical benefit of metformin in patients with idiopathic pulmonary fibrosis and type 2 diabetes mellitus: A national claims-based cohort analysis. Respir. Res..

[71] Tzouvelekis A., Tzilas V., Dassiou M., Bouros D. (2018). Metformin in Idiopathic Pulmonary Fibrosis “Seeking the Holy-Grail through Drug-Repositioning”. Respiration.

[72] Spagnolo P., Kreuter M., Maher T.M., Wuyts W., Bonella F., Corte T.J., Kopf S., Weycker D., Kirchgaessler K.U., Ryerson C.J. (2018). Metformin Does Not Affect Clinically Relevant Outcomes in Patients with Idiopathic Pulmonary Fibrosis. Respiration.

[73] Dixon A.E., Peters U. (2018). The effect of obesity on lung function. Expert Rev. Respir. Med..

[74] Sekine A., Wasamoto S., Hagiwara E., Yamakawa H., Ikeda S., Okabayashi H., Oda T., Okuda R., Kitamura H., Baba T. (2021). Beneficial impact of weight loss on respiratory function in interstitial lung disease patients with obesity. Respir. Investig..

[75] Okada-Iwabu M., Yamauchi T., Iwabu M., Honma T., Hamagami K.-i., Matsuda K., Yamaguchi M., Tanabe H., Kimura-Someya T., Shirouzu M. (2013). A small-molecule AdipoR agonist for type 2 diabetes and short life in obesity. Nature.

[76] Fang F., Liu L., Yang Y., Tamaki Z., Wei J., Marangoni R.G., Bhattacharyya S., Summer R.S., Ye B., Varga J. (2012). The adipokine adiponectin has potent anti-fibrotic effects mediated via adenosine monophosphate-activated protein kinase: Novel target for fibrosis therapy. Arthritis. Res. Ther..

[77] Zhang Y.Z., Zhang Y.L., Huang Q., Huang C., Jiang Z.L., Cai F., Shen J.F. (2019). AdipoRon Alleviates Free Fatty Acid-Induced Myocardial Cell Injury Via Suppressing Nlrp3 Inflammasome Activation. Diabetes Metab. Syndr. Obes..

[78] Rosen C.J. (2023). Antagonizing the Leptin Receptor in Obesity. N. Engl. J. Med..

[79] Wu D., Wang Z., Wang K., Wang Y., Wang T. (2024). The association between adipokines and pulmonary diseases: A mendelian randomization study. BMC Pulm. Med..

[80] Oh J.H., Chae G., Song J.W. (2024). Blood lipid profiles as a prognostic biomarker in idiopathic pulmonary fibrosis. Respir. Res..

[81] Barochia A.V., Kaler M., Weir N., Gordon E.M., Figueroa D.M., Yao X., Lemma WoldeHanna M., Sampson M., Remaley A.T., Grant G. (2021). Serum levels of small HDL particles are negatively correlated with death or lung transplantation in an observational study of idiopathic pulmonary fibrosis. Eur. Respir. J..

[82] Cai W., Zhang H., Li Z., Cai M., Chen P., Guo N., Song X. (2025). Potential biomarkers of idiopathic pulmonary fibrosis: Metabonomics driven lipid profiling. J. Transl. Med..

